# Supplementary material for: Application of Physiologically Based Pharmacokinetic Modeling to Predict Maternal Pharmacokinetics and Fetal Exposure to Oxcarbazepine
Source: Pharmaceutics. 2022 Nov 3;14(11):2367. doi: 10.3390/pharmaceutics14112367 (PMC9693517; doi:10.3390/pharmaceutics14112367)
Supplement: Supplementary file 1 [file pharmaceutics-14-02367-s001.zip › pharmaceutics-1923896-supplementary.pdf]

Supplemental Material

Application of Physiologically Based Pharmacokinetic Modeling to Predict Maternal Pharmacokinetics and Fetal Exposure to Oxcarbazepine

Lixia He[ǂ], Meng Ke[ǂ], Wanhong Wu, Jiarui Chen, Guimu Guo, Rongfang Lin, Pinfang Huang, Cuihong Lin*

***Corresponding Author**:
Cuihong Lin
Department of Pharmacy, The First Affiliated Hospital of Fujian Medical University, 20 Cha Zhong M. Rd, Fuzhou 350005, People's Republic of China.
Telephone: 86-591-8798 1331
Fax: 86-591-8798 1331
*E-mail:*lin1974@fjmu.edu.cn

TableS1. List of reported clinical studies in pregnancy that were used for modeling.

reference	Patient Population Number	weight(kg)	age(year)	Trimester	Dose(mg)
Mazzucchelli,I.et al.[1]	PG:5	54-62	22-34	P1;P2;P3	150-1500mg/d
Yin,X.et al.[2]	PG:56<br>WWE:53	NR	NR	P1;P2;P3	600-1050mg/d

NP = non-pregnant; P1 = first trimester; P2 = second trimester; P3 = third trimester; PG = pregnant;WWE: women with epilepsy.

Table S2. The expression concentrations of UGT1A9 and UGT2B7 during different periods, across organs and tissues (mainly), and the difference in renal blood flow between nonpregnant and pregnant women.

**enzymes**	UGT1A9(μmol/l)			UGT2B7(μmol/l)
	Kidey-intracellular	Kidey-tissue	liver periportal -intracellular	liver periportal -intracellular
nonpregnancy	1.77		0.15	1.71
1st trimester	1.78	1.32	0.15	1.7
2nd trimester	1.77	1.32	0.15	1.68
3rd trimester	1.78	1.32	0.15	1.64
**renal clearances**	plasma clearance(ml/min/kg)			
nonpregnancy	0.27			
1st trimester	5			
2nd trimester	5			
3rd trimester	5			

Supplementary Workflow Figures:

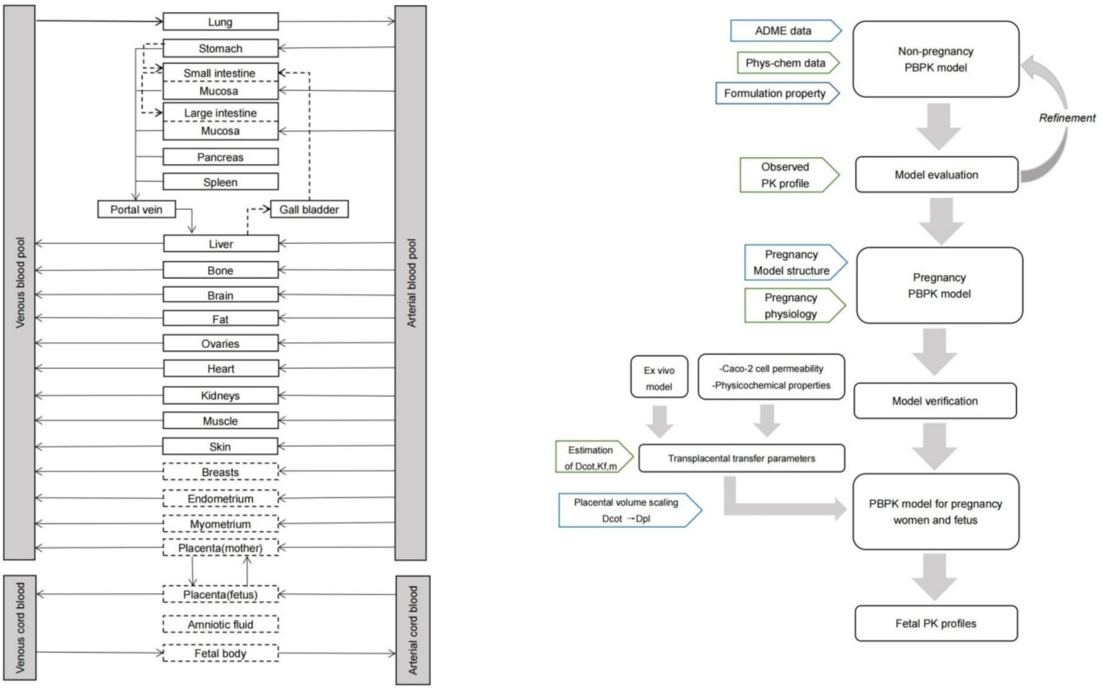

Figure S1. Structure of the pregnancy PBPK model and fetal-maternal physiologically based pharmacokinetic (f-m PBPK) model. On the left is the structure of the pregnancy PBPK model. Thick arrows indicate drug transport through blood flow, and thin arrows indicate drug transport through other routes (e.g., gastrointestinal passages, biliary excretion through the gallbladder, and diffusive transfer within the placenta). Compartments that only belong to the structure of the pregnancy PBPK model are shown in italics, with dashed borders and dashed arrows indicating drug transfer through the bloodstream. On the right is a schematic representation of the work in development and evaluation of the f-m PBPK model.[3-5]D_{cot}, transcotyledon passive diffusion clearance; D_{pl}, passive fusion clearance through placenta; $K_{f,m}$, partition between fetal and maternal compartments; PBPK, physiologically based pharmacokinetics; PK, pharmacokinetics.

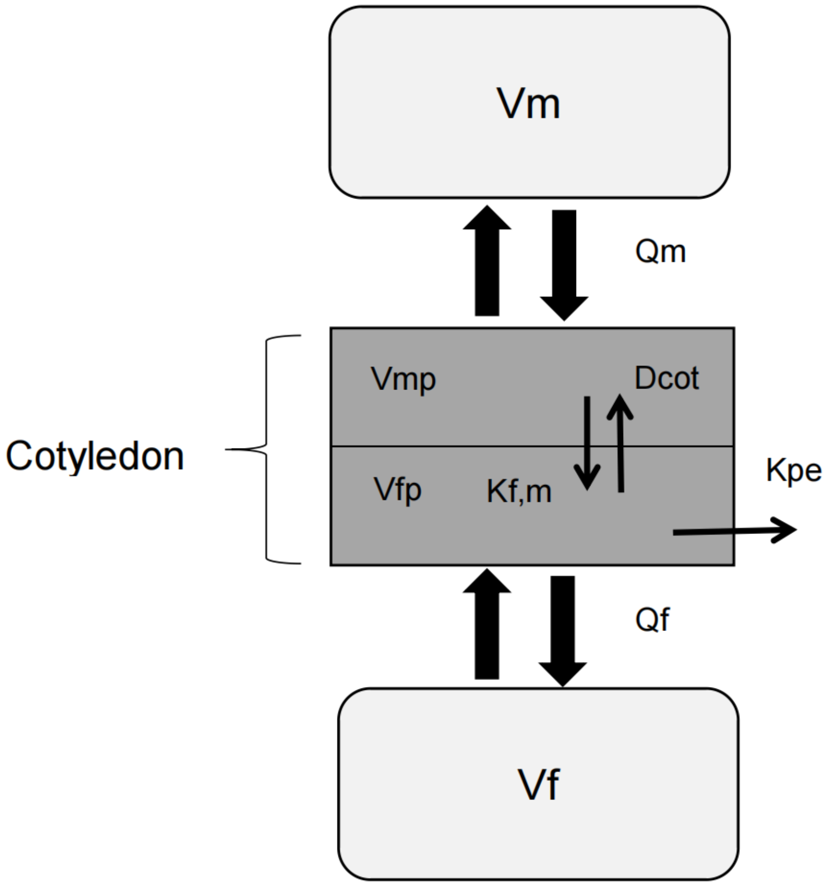

Figure S2. Schematic representation of the ex vivo cotyledon perfusion model.[6-7]

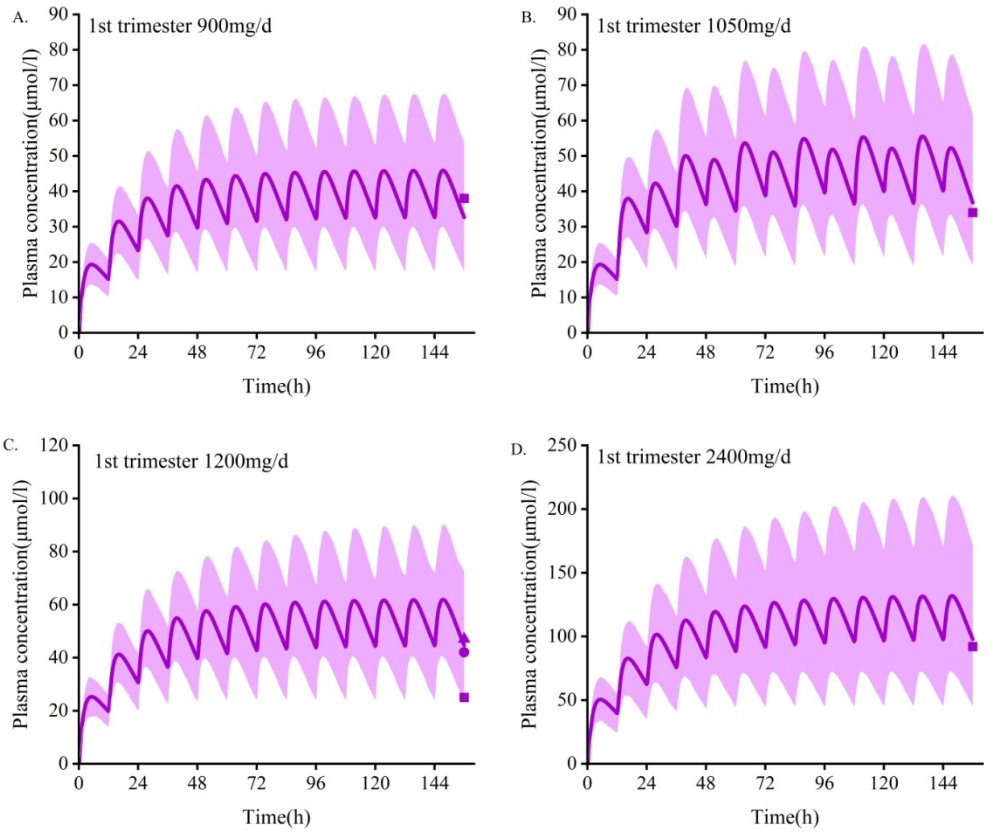

Figure S3. Median plasma concentration of the population taking different doses of OXC in the first trimester of pregnancy predicted by population PBPK simulation (A–D) is shown as a dark line, and the shaded area represents the 5th to 95th prediction range. OXC, oxcarbazepine.

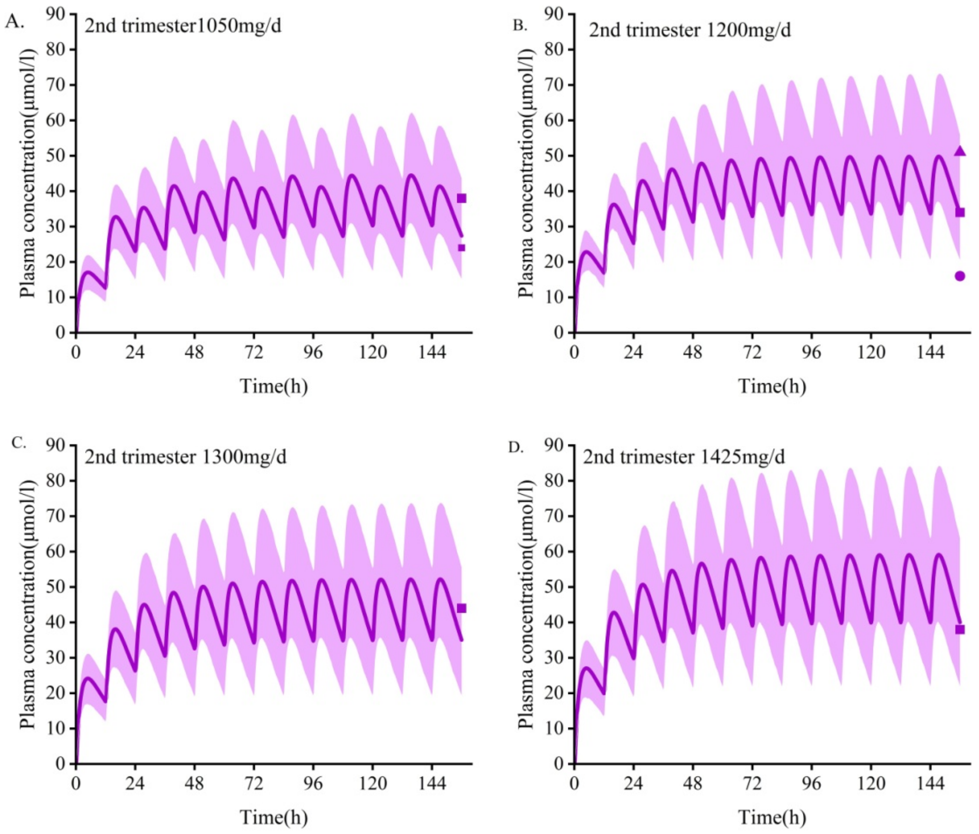

Figure S4. Median plasma concentration of the population taking different doses of OXC in the second trimester of pregnancy predicted by population PBPK simulation (A–D) is shown as a dark line, and the shaded area represents the 5th to 95th prediction range.

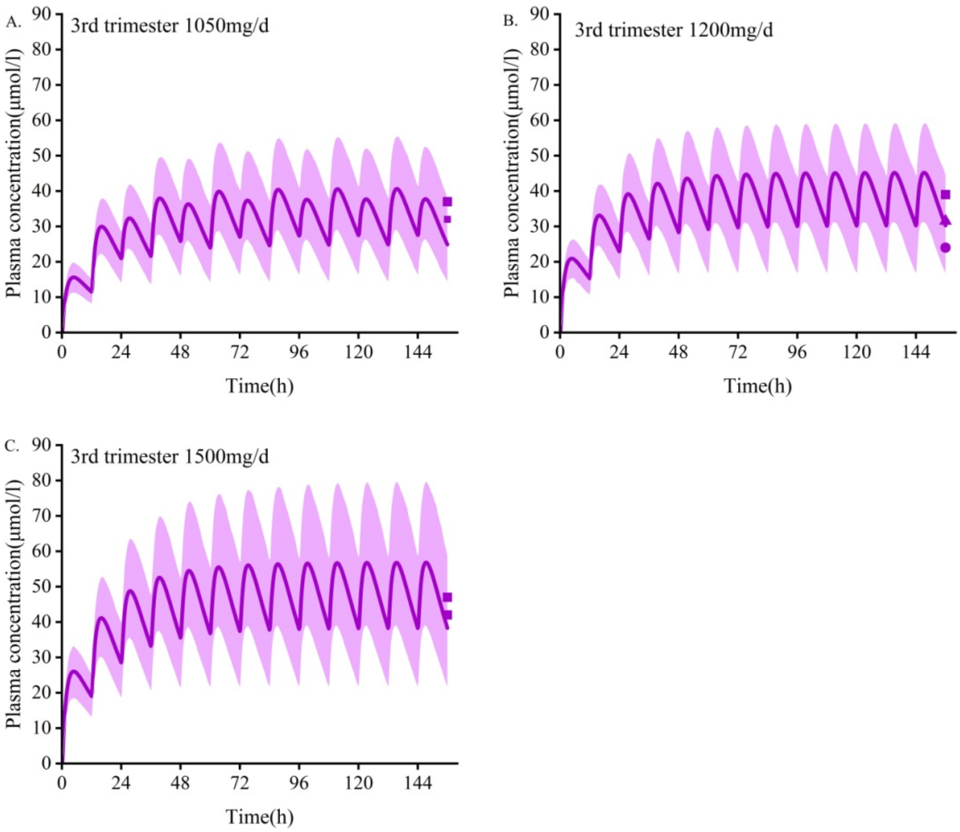

Figure S5. Median plasma concentration of the population taking different doses of OXC in the third trimester of pregnancy predicted by population PBPK simulation (A–C) is shown as a dark line, and the shaded area represents the 5th to 95th prediction range.

References

1. Mazzucchelli, I.; Onat, F.Y.; Ozkara,C.; Atakli,D.; Specchio,L.M.; Neve,A.L.; Gatti,G.; Perucca E. Changes in the Disposition of Oxcarbazepine and Its Metabolites during Pregnancy and the Puerperium. *Epilepsia* **2006**,*47*,504-509,doi: 10.1111/j.1528-1167.2006.00459.x.
2. Yin X, Liu Y, Guo Y, Zhao L, Li G, Tan X. Pharmacokinetic changes for newer antiepileptic drugs and seizure control during pregnancy. CNS Neurosci Ther. 2022, 28(5):658-666. doi: 10.1111/cns.13796. Epub 2022 Jan 17. PMID: 35037389; PMCID: PMC8981429.
3. Dallmann, A.; Solodenko, J.; Ince, I.; Eissing, T. Applied Concepts in PBPK Modeling: How to Extend an Open Systems Pharmacology Model to the Special Population of Pregnant Women. *CPT Pharmacometrics Syst Pharmacol* **2018** ,*7*,419-431,doi: 10.1002/psp4.12300.
4. Song,L.;Yu,Z.;Xu,Y.;Li,X.;Liu,X.;Liu,D.;Zhou,T. Preliminary Physiologically Based Pharmacokinetic Modeling of Renally Cleared Drugs in Chinese Pregnant Women. *Biopharm Drug Dispos* **2020**,*41*,248-267,doi: 10.1002/bdd.2243.
5. Mian,P.;Allegaert,K.;Conings,S.;Annaert,P.;Tibboel,D.;Pfister,M.;van Calsteren,K.;van den Anker,J.N.;Dallmann,A. Integration of Placental Transfer in a Fetal-Maternal Physiologically Based Pharmacokinetic Model to Characterize Acetaminophen Exposure and Metabolic Clearance in the Fetus. *Clin Pharmacokinet* **2020** ,*59*,911-925,doi: 10.1007/s40262-020-00861-7.
6. De Sousa Mendes, M.; Hirt, D.; Vinot, C.; Valade, E.; Lui, G.; Pressiat, C.; Bouazza, N.; Foissac, F.; Blanche, S.; Lê,M.P.; Peytavin,G. et al. Prediction of Human Fetal Pharmacokinetics Using Ex Vivo Human Placenta Perfusion Studies and Physiologically Based Models. *Br J Clin Pharmacol* **2016**,*81*,646-57,doi: 10.1111/bcp.12815.
7. Mian, P.; Allegaert, K.; Conings, S.; Annaert, P.; Tibboel, D.; Pfister, M.; van Calsteren, K.; van den Anker, J.N.; Dallmann, A. Integration of Placental

Transfer in a Fetal-Maternal Physiologically Based Pharmacokinetic Model to Characterize Acetaminophen Exposure and Metabolic Clearance in the Fetus. *Clin Pharmacokinet* **2020**,*59*,911-925,doi: 10.1007/s40262-020-00861-7.
